# Prevalence and risk factors for childhood anemia in Rwanda: Using Rwandan demographic and health survey 2019–2020

**DOI:** 10.1002/puh2.159

**Published:** 2024-02-14

**Authors:** Henriette Usanzineza, Etienne Nsereko, Jean Pierre Niyitegeka, Aline Uwase, Jean de Dieu H. Tuyishime, Francois Xavier Sunday, Christian Mazimpaka, Jeanine Ahishakiye

**Affiliations:** ^1^ Department of Anesthesia School of Health Science College of Medicine and Health Sciences University of Rwanda Kigali Rwanda; ^2^ Department of Nutrition School of Public Health College of Medicine and Health Sciences University of Rwanda Kigali Rwanda; ^3^ IntraHealth International Kigali Rwanda

**Keywords:** anemia, determinants, factors associated, predictors, prevalence, risk factors, Rwanda DHS

## Abstract

**Introduction:**

Anemia in children is a significant health issue globally, with developing countries, notably Africa, being disproportionately affected. This condition can result in detrimental and irreversible impacts on a child's neurological development. Despite its relevance, research on anemia prevalence and risk factors in Rwandan children aged 6–23 months is limited. Our study aimed to ascertain the prevalence and potential risk factors associated with anemia in this defined population.

**Methods:**

This is a cross‐sectional study that used secondary data analysis on a weighed sample of 1247 children aged 6–23 months, sourced from the 2019–2020 Rwanda Demographic Health Survey. We used descriptive statistics and binary logistic regression to identify the links between anemia and various factors.

**Results:**

The study revealed a high prevalence of anemia at 52.79%. Among anemic children aged 6–23 months, most (52.82%) had mild anemia, 46.12% had moderate anemia, and 1.06% had severe anemia. Key contributors to childhood anemia included maternal anemia (adjusted odds ratio [AOR] = 1.62, 95% confidence intervals (95% CI): [1.11, 2.33]) and history of coughing in the 2 weeks before the survey (AOR = 1.42, 95% CI: [1.12, 1.81]). Vitamin A supplementation and antiparasitic medication were identified as protective factors (AOR = 0.70, 95% CI: [0.50, 0.97]) and (AOR = 0.59, 95% CI: [0.45, 0.77]), respectively.

**Conclusion:**

Given the high anemia prevalence among Rwandan children aged 6–23 months, targeted public health interventions are critically needed. A comprehensive strategy throughout the health system is essential for reducing childhood anemia, involving measures such as addressing maternal anemia, managing childhood illnesses, and enhancing existing interventions like vitamin A supplementation and deworming.

## Graphical abstract

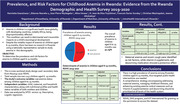

## INTRODUCTION

Anemia, a condition marked by insufficient hemoglobin levels in the blood, disproportionately affects children aged 6–23 months in developing countries, particularly in Sub‐Saharan Africa [1, 2, 3]. The susceptibility of this age group to anemia is largely due to their increased iron requirements for rapid growth and development [1, 2]. Iron deficiency is a significant contributor, accounting for more than half of all global anemia cases, and other nutritional deficiencies, such as vitamins A and B12, also play a role [4, 5]. Additionally, infections, such as malaria and intestinal parasites, can lead to anemia through inflammation and blood loss [5, 6, 7].

The ramifications of childhood anemia are profound, including cognitive and physical development delays, reduced immunity, and an increased risk of morbidity and mortality [1, 8–10]. Despite considerable efforts, such as iron supplementation, deworming, and the distribution of insecticide‐treated bed nets [11], anemia remains a critical public health concern, particularly in developing countries [12]. In 2019, the World Health Organization (WHO) disclosed that approximately 40% of children aged 6–59 months, totaling 269 million, were anemic, with the heaviest toll felt in Africa's 60.2% prevalence rate [13, 14]. Notably, children aged 6–23 months showed a particularly high prevalence rate of 70% in low‐ and lower middle‐income countries [15]. This high prevalence is significantly associated with various factors, such as family wealth, maternal anemia, maternal education, and low birth weight [15].

In response, the Rwandan government has launched initiatives targeting risk factors associated with anemia, including deworming, vitamin A supplementation, malaria prevention, agricultural initiatives, and improvements in feeding practices, education, and water, sanitation, and hygiene [16]. However, anemia remains a significant public health issue in Rwanda, with 37% of children aged 6–59 months experiencing some degree of anemia, especially among the 6–23‐month age group [17]. Despite the high prevalence and adverse impacts of anemia, there is a dearth of research on anemia in Rwandan children aged 6–23 months, underscoring the urgency to fill this knowledge gap. Our study, therefore, aimed to identify factors associated with anemia in this vulnerable age group to inform appropriate strategies and interventions to mitigate this critical public health concern.

## METHODS

### Source of data, study design, and sampling procedure

This study utilized secondary data from the recent available 2019–2020 Rwanda Demographic and Health Survey (RDHS), a population‐based cross‐sectional study design. The RDHS calculates key demographic and health indicators for the country using nationally representative samples [17]. The sample was chosen through a two‐stage stratified sampling method in order to estimate the key demographic and health indicators at the national level, each in 5 provinces, and 30 districts, including both rural and urban regions. Stepwise, clusters were drawn from a master sampling frame made up of all villages, with a probability relative to the number of households in each village, and 500 enumeration areas (EAs) were selected. Secondary surveyors mapped and systematically sampled the households within the selected EAs. Twenty‐six households were chosen from each sample EA, totaling 13,000 households in the sample [17].

After the random selection of households, data were collected from 15–49‐year‐old women and 6–59‐month‐old children who have stayed the night at home prior the survey. Five questionnaires were utilized to gather data on basic demographic, household characteristics, anthropometry measures, and other biomarker results like anemia and malaria testing. Mothers and other primary caregivers were interviewed regarding the health of their children (aged 6–59 months) and provided consent for blood draws from both children and women (aged 15–49 years) to measure hemoglobin levels. Finger prick blood was tested for hemoglobin levels (or heal prick in case of children within an age range of 6–11 months). On site, a portable HemoCue analyzer that runs on battery was used to measure hemoglobin level. Hemoglobin levels were successfully measured to 3525 children within the ages of 6–59 months, including 1247 infants and young children 6–23 months, from the involved household. However, hemoglobin levels were available for 1245 children.

The Kids Records dataset was utilized in our analysis. This dataset includes all information related to child, maternal, and household characteristics, feeding practice, child and maternal nutritional profiles, and health status along with anemia level for every kid of women who was questioned and was born during the preceding 5 years.

### Study variables

#### Dependent variable

The study's outcome variable was anemia status among children aged 6–23 months. Blood was tested for hemoglobin concentration levels measured in g/dL.

According to WHO guidelines for children aged 6–23 months, anemia is defined as hemoglobin concentrations less than 11.0 g/dL. For analysis purposes, anemic cases (hemoglobin <11 g/dL) were assigned a “yes” label, encompassing all severities (mild, moderate, and severe), whereas nonanemic cases (hemoglobin ≥11 g/dL) were labeled “no”.

#### Independent variable

Based on the existing literature [3], we extracted a specific set of variables relating to child, maternal, and household characteristics, along with nutritional profiles and health status variables of both mothers and children from the RDHS dataset for utilization in this study.

The selected variables were subdivided into:

*Child and maternal characteristics* that included sex of child (male vs. female), child age in months starting from 6 to 23 months (successively grouped into 6–8; 9–11, 12–17, and 18–23 months). Age of mother (in year) categorized (<20, 20–29, 30–39, ≥40), education of mother (no formal education, primary education, secondary and higher), maternal working status (not working vs. working), marital status (never married, current married/in union and divorced/separated/widow).
*Household characteristics* comprised: children under five sleeping under a mosquito net (no, yes), wealth index categories (as poorest, poorer, middle, richer, and richest); place of residence (urban or rural), toilet facilities and sources of drinking water (both broken down into binary variables, improved and not improved) as defined by the WHO/UNICEF Joint Monitoring Program for water supply and sanitation [18], and stool disposal (appropriate, not appropriate) [18].
*Feeding characteristics*: Current breastfeeding (no, yes), minimum dietary diversity (adequate dietary diversity represents the proportion of those who receive from five or more food groups out of eight food groups in the preceding 24 h, and low dietary diversity refers to less than five groups). Dietary diversity was assessed by choosing from a list of eight food groups, which included *breast milk*; *grains, roots, and tubers*; *legumes and nuts*; *dairy products*, for example, milk, yogurt, and cheese; *flesh foods* (meat, fish, poultry, and liver/organ meat); *eggs*; *vitamin A‐rich fruits and vegetables*; and *other fruits and vegetables* [17].
*Child/maternal nutritional characteristics*: Vitamin A supplementation within the 6 months preceding the survey (no, yes). Stunting status, defined as kids with a height‐for‐age *Z*‐score below minus two standard deviations (−2 SD) from the reference population mean [17, 18], (not stunted, stunted); underweight status (referring to offspring with a weight‐for‐age less than minus two standard deviations “−2 SD” from the mean of the reference population) [17, 18] (no, yes); wasting (defined as young kid with weight‐for‐height *Z*‐score less than minus two standard deviations (−2 SD) from the reference population mean [17, 18] (no, yes); maternal anemia (anemic, not anemic) and maternal body mass index (underweight [<18.5 kg/m^2^], normal weight [18.5–24.9 kg/m^2^], overweight and obese ≥25 kg/m^2^) [18].
*Health status characteristics*: Recent occurrence of fever, diarrhea, and cough within the last 2 weeks preceding the survey (no, yes); status on deworming medication (asked if mother provided intestinal parasite medication in the last 6 months; no, yes).

## DATA ANALYSIS

Before carrying out any analysis, a sample weight was applied to ensure the representativeness of survey result to entire country. The data preparation included recording the variables of interest.

Descriptive statistics, including frequencies and percentages for categorical variables, were used to describe the characteristics of the study participants and determine the prevalence of anemia. To explore the relationship between potential risk factors and childhood anemia, a bivariate analysis was performed. Pearson's chi‐squared (*χ* ^2^) test was then used to examine the significance of associations between anemia and each explanatory variable. Subsequently, any variable with a *p*‐value below 0.1 was included in the multivariable logistic regression model. The final multivariable model (adjusted model) was developed using backward‐stepwise regression, and variables were ranked according to the level of impact on childhood anemia based on previous studies [19]. Variables were removed one at a time if the *p*‐value >0.05, starting with variable with the highest *p*‐value, stopping when all remaining variables were statistically significant. Child age and deworming were correlated (*r* > 0.6); however, we only included deworming as it had a biological plausibility than age (age is a nonmodifiable risk factor). A *p*‐value <0.05 was considered statistically significant. Results were presented using odds ratio with the corresponding 95% confidence intervals (CIs). All analysis was performed using SPSS 25.

### Ethical considerations

This study utilized the 2019–2020 RDHS database, an international survey executed every 5 years and approved by Rwanda's Institutional Review Board. Permission to access and use the dataset was granted by ICF International after topic registration and submission via their website. The study received ethical approval from the IRB_CMHS committee (Reference No. 373/CMHSIRB/2022) post‐ICF authorization.

## RESULTS

### Child, maternal, and household characteristics

Table 1 presents child, maternal, and household characteristics for 1247 children aged 6–23 months and their mothers. Findings show 51.60% of children were male, with the most represented age groups being 12–17 and 12–23 months (32.50% and 32.20%, respectively). Most mothers were aged 30–39 (44.80%) with primary education (63.33%), in a union (83.30%), and employed (72.60%). Regarding household factors, 66.90% of children under five slept under mosquito nets, 83.20% lived in rural areas, 21.90% were in the poorest wealth index, 78.90% had access to improved water, 75.20% had improved sanitation, and 91.30% had appropriate stool disposal.

**TABLE 1:** Child, maternal, and household characteristic, Rwanda Demographic and Health Survey 2019–2020 (*n* = 1247).

Variables	Frequency	Percentage
**Sex**		
Male	644	51.60
Female	603	48.40
**Child age**		
6–8 months	218	17.40
9–11 months	223	17.90
12–17 months	406	32.50
18–23 months	401	32.20
**Mother's age**		
<20 years	30	2.40
20–29 years	517	41.50
30–39 years	559	44.80
40–49 years	141	11.30
**Mother's education level**		
No education	122	9.74
Primary	790	63.33
Secondary	277	22.20
Higher	59	4.73
**Maternal working status**		
Not working	342	27.40
Working	905	72.60
**Current marital status**		
Never married	130	10.43
Currently married/in union	1039	83.33
Divorced/separated/widow	78	6.24
**Sleeping under mosquito net**		
No	413	33.10
Yes	834	66.90
**Type of residence (*n* = 1246)**		
Urban	210	16.80
Rural	1038	83.20
**Wealth index**		
Poorest	274	21.90
Poorer	272	21.80
Middle	230	18.40
Richer	261	20.90
Richest	211	16.90
**Source of drinking water (*n* = 1228)**		
Improved	969	78.90
Not improved	259	21.10
**Type of toilet facility (*n* = 1197)**		
Not improved	296	24.80
Improved	901	75.20
**Stool disposal**		
Appropriate	1139	91.30
Not appropriate	109	8.70

### Child/maternal nutritional profile and health characteristics

Table 2 presents dietary and health profiles of children and their mothers. Majority (92.2%) of children were breastfed, though 66.10% had poor dietary diversity. Overall, 80.70% received vitamin A supplements. Stunting, wasting, and underweight prevalence were 31.10%, 1.50%, and 7.50%, respectively, whereas 11.30% of mothers were anemic. Overall, 24.80%–36.70% of children experienced diarrhea, fever, or cough, and 60.90% received deworming medication.

**TABLE 2:** Child/maternal nutritional profile and health characteristics, Rwanda Demographic and Health Survey 2019–2020 (*n* = 1247).

Variables	Frequency	Percentage
**Breastfeeding status**		
No	96	7.70
Yes	1151	92.30
**MDD**		
Low DD	824	66.10
Adequate DD	423	33.90
**Vitamin A supplements (*n* = 1246)**		
No	240	19.30
Yes	1006	80.70
**Stunting status (*n* = 1246)**		
Stunted	384	30.80
Not stunted	862	69.20
**Underweight status (*n* = 1246)**		
Yes	93	7.50
No	1152	92.50
**Wasted (*n* = 1246)**		
Wasted	19	1.50
Not wasted	1226	98.50
**Maternal BMI kg/m^2^ (*n* = 1246)**		
Underweight (<18.5 kg/m^2^)	51	4.10
Normal weight (18.5–24.9 kg/m^2^)	866	69.60
Overweight and obesity (≥25 kg/m^2^)	328	26.30
**Maternal anemia**		
Not anemic	1103	88.70
Anemic	141	11.30
**Recent episodes of fever**		
No	908	72.80
Yes	340	27.20
**Recent episodes of diarrhea**		
No	938	75.20
Yes	310	24.80
**Recent episodes of cough**		
No	790	63.30
Yes	457	36.70
**Deworming**		
No	491	39.30
Yes	757	60.70

Abbreviations: BMI, body mass index; DD, dietary diversity; MDD, minimum dietary diversity.

### Prevalence of anemia among children aged 6–23 months

**FIGURE 1:**
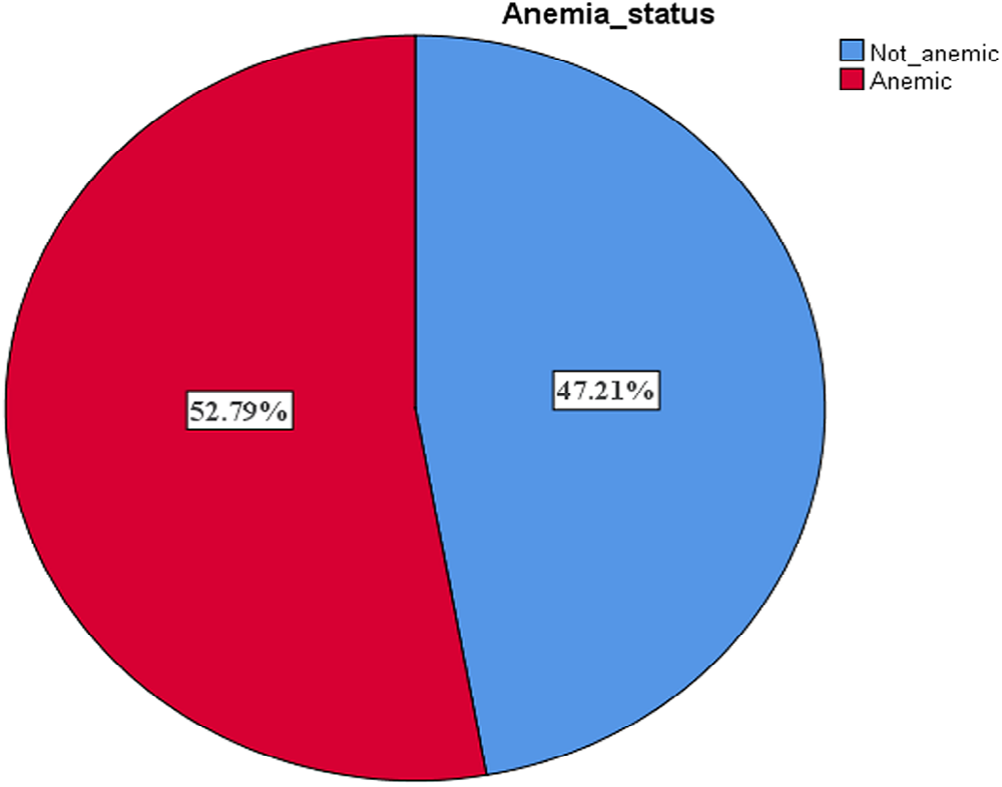
Prevalence of anemia among children aged 6–23 months, Rwanda Demographic and Health Survey 2019–2020.

**FIGURE 2:**
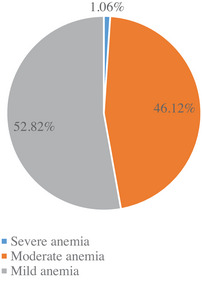
Distribution of anemia by severity among children aged 6–23 months, Rwanda Demographic and Health Survey 2019–2020.

Figure 1 depicts the overall prevalence of anemia in children aged 6–23 months was 52.79% (657/1245). Regarding the severity classification of anemia, most cases of anemia were in the mild range 52.82%, followed by moderate anemia 46.12%, and the severe anemia category contributed the least 1.06% (Figure 2).

### Association of anemia and child, maternal, and household characteristics

Table 3 displays the association between anemia and child/maternal and household characteristics. Findings show that only children age categories (*p* < 0.001) and stool disposal (*p* = 0.021) correlate with anemia.

**TABLE 3:** Bivariate analysis of anemia and child/maternal and household characteristics among children aged 6–23 months, Rwanda Demographic and Health Survey 2019–2020 (*n* = 1245).

Variables	Anemic	Not anemic	*p*‐Value
**Sex**			0.848
Male	341 (53.00)	302 (47.00)	
Female	316 (52.50)	286 (75.50)	
**Child age**			0.001
6–8 months	151 (69.60)	66 (30.40)	
9–11 months	143 (64.70)	78 (35.30)	
12–17 months	207 (51.00)	199 (49.00)	
18–23 months	155 (38.75)	245 (61.25)	
**Mother's age**			0.118
<20 years	19 (65.50)	10 (34.50)	
20–29 years	267 (51.70)	249 (48.30)	
30–39 years	306 (54.80)	252 (45.20)	
40–49 years	64 (45.70)	76 (54.30)	
**Mother's education level**			0.520
No education	68 (55.70)	54 (44.30)	
Primary	418 (53.00)	371 (47.00)	
Secondary	146 (52.70)	131 (47.30)	
Higher	25 (43.90)	32 (56.10)	
**Maternal working status**			0.894
Not working	181 (53.10)	160 (46.90)	
Working	476 (52.70)	428 (47.30)	
**Current marital status**			0.822
Never married	66 (50.80)	64 (49.20)	
Currently married/in union	549 (52.90)	488 (47.10)	
Divorced/separated/widow	43 (55.10)	35 (44.90)	
**Sleeping under mosquito net**			0.141
No	230 (55.70)	183 (44.30)	
Yes	426 (51.30)	405 (48.70)	
**Type of residence**			0.907
Urban	109 (52.40)	99 (47.60)	
Rural	548 (52.80)	489 (47.20)	
**Wealth index**			0.301
Poorest	156 (57.10)	117 (42.90)	
Poorer	138 (50.90)	133 (49.10)	
Middle	123 (53.30)	107 (46.50)	
Richer	140 (53.80)	120 (46.20)	
Richest	100 (47.60)	110 (52.40)	
**Source of drinking water**			0.400
Improved	517 (53.50)	449 (46.50)	
Not improved	131 (50.6)	128 (49.40)	
**Type of toilet**			0.681
Not improved	151 (51.00)	145 (49.00)	
Improved	471 (52.4)	428 (47.6)	
**Stool disposal**			0.021
Appropriate	588 (51.80)	548 (48.20)	
Not appropriate	69 (63.30)	40 (36.70)	

### Association of anemia and child/maternal nutritional profile and health characteristics

Table 4 presents the relationship between anemia status and child/maternal nutritional profile and health characteristics. Findings reveal that children who did not meet the minimum dietary diversity level (*p* = 0.024), children who did not receive vitamin A supplement (*p* < 0.001) in the last 6 weeks prior to the survey, and children from anemic mothers (*p* = 0.012) are associated with anemia. Similarly, anemia was considerably higher among children who got cough in 2 weeks prior the survey (*p* < 0.001) and those who did not took the deworming medicate (*p* < 0.001).

**TABLE 4:** Bivariate analysis of anemia status by child/maternal nutritional profile and health characteristics among children aged 6–23 months, Rwanda Demographic and Health Survey 2019–2020 (*n* = 1245).

Variables	Anemic	Not anemic	*p*‐Value
**Breastfeeding status**			0.317
No	46 (47.90)	50 (52.10)	
Yes	611 (53.20)	537 (46.80)	
**MDD**			0.021
Low DD	454 (55.10)	370 (44.90)	
Adequate DD	203 (48.20)	218 (51.80)	
**Vitamin A supplements**			<0.001
No	158 (65.80)	82 (43.20)	
Yes	499 (49.70)	505 (50.30)	
**Stunting status**			0.283
Stunted	211 (55.10)	172 (44.90)	
Not stunted	446 (51.80)	415 (48.20)	
**Underweight status**			0.089
Yes	57 (61.30)	36 (38.70)	
No	600 (52.10)	551 (47.90)	
**Wasted**			0.652
Wasted	11 (57.90)	8 (42.10)	
Not wasted	646 (52.70)	580 (47.30)	
**Maternal BMI kg/m^2^**			0.227
Underweight (<18.5 kg/m^2^)	28 (54.9)	23 (45.10)	
Normal weight (18.5–24.9 kg/m^2^)	470 (54.3)	396 (45.70)	
Overweight and obesity (≥25 kg/m^2^)	160 (48.8)	168 (51.20)	
**Maternal anemia**			0.012
Not anemic	569 (51.60)	534 (48.40)	
Anemic	88 (62.90)	52 (37.10)	
**Recent episodes of fever**			0.402
No	471 (52.00)	434 (48.00)	
Yes	186 (54.70)	154 (45.30)	
**Recent episodes of diarrhea**			0.078
No	480 (51.30)	455 (48.70)	
Yes	177 (57.10)	133 (42.90)	
**Recent episodes of cough**			0.001
No	388 (49.30)	399 (50.70)	
Yes	269 (58.90)	188 (41.10)	
**Deworming**			<0.001
No	307 (62.70)	183 (37.30)	
Yes	350 (46.40)	404 (53.60)	

Abbreviations: BMI, body mass index; DD, dietary diversity; MDD, minimum dietary diversity.

### Determinants of anemia among children aged 6–23 months

Table 5 presents the multivariate logistic regression analysis of factors influencing anemia in children aged 6–23 months. The results suggest children receiving Vitamin A supplements were less likely to be anemic (adjusted odds ratio [AOR] = 0.70, 95% CI: [0.50, 0.97], *p* = 0.036). Children of anemic mothers had a higher anemia risk (AOR = 1.61, 95% CI: [1.11, 2.33], *p* = 0.011). Health status‐wise, children having a recent cough were more likely to be anemic (AOR = 1.43, 95% CI: [1.13, 1.81], *p* = 0.003), whereas those receiving deworming medication had reduced anemia risk (AOR = 0.59, 95% CI: [0.45, 0.77], *p* < 0.001).

**TABLE 5:** Multivariate logistic regression of the determinants of anemia in children aged 6–23 months, Rwanda Demographic and Health Survey 2019–2020.

	Full model			Adjusted model		
	OR	[95% CI]	*p*‐Value	OR	[95% CI]	*p*‐Value
**Stool disposal**						
Appropriate	1					
Not appropriate	1.24	[0.81, 1.89]	0.311			
**MDD_**						
Low DD	1					
Adequate DD	0.81	[0.64, 1.04]	0.100			
**Vitamin A supplement**						
No	1			1		
Yes	0.70	[0.50, 0.98]	0.041	0.70	[0.50, 0.97]	0.036
**Underweight status**						
Yes	1					
No	0.84	[0.53, 1.31]	0.447			
**Maternal anemia**						
Not anemic	1			1		
Anemic	1.61	[1.11,2.33]	0.011	1.61	[1.11, 2.33]	0.011
**Recent episodes of diarrhea**						
No	1					
Yes	1.09	[0.83,1.44]	0.498			
**Recent episode of cough**						
No	1			1		
Yes	1.38	[ 1.08,1.76]	0.010	1.43	[1.13, 1.81]	0.003
**Deworming**						
No	1			1		
Yes	0.60	[0.46, 0.79]	<0.001	0.59	[0.45, 0.77]	<0.001

*Note*: Statistically significant association (*p* < 0.05).

Abbreviations: 1, reference; AOR, adjusted odds ratio; CI, confidence interval; DD, dietary diversity; MDD, minimum dietary diversity; OR, odds ratio.

## DISCUSSION

### Prevalence of anemia among children aged 6–23 months

The prevalence of anemia among Rwandan children aged 6–23 months was found to be 52.79%, indicating a severe public health issue per WHO classification [1]. The high prevalence may be due to the fact that only 60.70% of children received deworming medication and 80.70% vitamin A supplements 6 weeks prior to the survey. Furthermore, only 66.90% of children under five slept under mosquito nets. Given the prevalence of malaria (2.70%) among this age group, as per the DHS 2019–2020, this could contribute to anemia due to hemolysis of erythrocytes [20]. This rate is lower than studies from Ethiopia (72.3%) [9, 21], Myanmar (76%) [22], 32 Sub‐Saharan African nations (76.6%) [3], and 50 low‐ to lower middle‐income countries (70%) [15]. High prevalence in these regions is likely due to increased susceptibility to infectious diseases affecting vitamin absorption, leading to anemia [3]. However, our anemia prevalence was higher than that reported in Ghana (46%) and significantly higher than that in China (27%) [23]. The lower rates in these regions may be due to socioeconomic and educational development, alongside effective anemia reduction policies. High anemia prevalence in Rwandan children aged 6–23 months necessitates a comprehensive and multifaceted approach to address this public health concern. Thus, a national strategy for the prevention and control of anemia should be developed, targeting children aged 6–23 months and other vulnerable groups like children under 5, breastfeeding mothers, and pregnant women. Additionally, the implementation of routine hemoglobin screening for all children aged 6–23 months as part of their regular child healthcare visits will enable early detection, prompt intervention, and treatment of anemia, ultimately reducing the overall prevalence of the condition.

### Determinants of anemia in children aged 6–23 months

This study found children born to mothers with anemia were likely to develop anemia, possibly due to shared socioeconomic and dietary conditions [15, 24]. Such children may have limited access to nutritional food as maternal insufficient nutrition intake often reflects in their offspring [15, 25]. This is consistent with studies from Ethiopia, Myanmar, Southern African countries, and 50 nations with low‐ and lower middle incomes [15, 21, 22, 26, 27]. Anemia in children can be significantly prevented by prioritizing the reduction of maternal anemia. This can be achieved by promoting and supporting improved maternal and child nutrition through programs focusing on iron supplementation for pregnant women, breastfeeding promotion, complementary feeding practices, and dietary diversification. By implementing these interventions during the 1000‐day window, a time frame shaping children's health from pregnancy to age two, we can significantly reduce the risk of anemia in children.

Children with a recent cough were also found to be more prone to anemia. This could be due to the relationship between anemia and lower respiratory infections, which affect iron usage and immune system function, increasing susceptibility to common illnesses [28, 29]. This aligns with studies from Rwanda and Tanzania [30, 31, 32] but contrasts with findings from India [29]. Thus, strengthening child care services like early recognition and treatment of childhood illnesses like cough could help reduce anemia in children.

Children who received vitamin A supplements were less likely to develop anemia, as vitamin A improves hematopoiesis and iron mobilization and has an immune modulator effect [1, 33]. This supports an Ethiopian study [34] but contradicts studies from Namibia and Peru [35, 36]. Further, deworming medication was found to protect against anemia, likely due to its effect on soil‐transmitted helminths that cause iron and protein loss [37]. This is consistent with studies from Sub‐Saharan Africa and Bangladesh [38, 39], but at odds with studies from Ethiopia, Rwanda, and Namibia [13, 41]. To further enhance the reduction of childhood anemia, the Rwandan government and other stakeholders should strengthen existing interventions like deworming and vitamin A supplements by increasing country‐wide coverage and compliance rate among children aged 6–23 months. Lastly, the study found no significant association between anemia and minimum dietary diversity level in multivariate analysis. This may be due to the DHS's short‐term dietary assessment, which may not accurately represent typical dietary patterns [22]. This is similar to a study from Bangladesh [40] but differs from studies from low‐ and middle‐income countries and Ghana [15, 41].

This study has certain limitations that should be considered. The cross‐sectional design used in this study limits our ability to definitively determine cause‐and‐effect relationships between variables. Additionally, self‐reporting and retrospective data collection methods may have introduced recall bias and social desirability bias. Lack of data on specific causes of anemia and additional biomarkers was another limitation.

## CONCLUSION

The findings of this study emphasize the severity of anemia among Rwandan children aged 6–23 months, despite the implementation of various interventions. Maternal anemia and recent cough were identified as risk factors, whereas vitamin A supplements and deworming medication showed a protective effect. Public health approaches and interventions aimed at preventing and reducing childhood anemia must be developed across various government levels. These efforts should prioritize the reduction of maternal anemia, the strengthening of child care services, and the enhancement of coverage and compliance with existing interventions, such as deworming and vitamin A supplementation.

## AUTHOR CONTRIBUTIONS

Conceptualization; methodology; writing—review and editing; formal analysis; project administration; writing—original draft: Henriette Usanzineza. Conceptualization; writing—review and editing; methodology; writing—original draft; formal analysis: Etienne Nsereko. Writing—original draft; conceptualization; methodology; validation; formal analysis: Jean Pierre Niyitegeka. Writing—original draft; writing—review and editing; methodology: Aline Uwase and Christian Mazimpaka. Conceptualization; writing—original draft; writing—review and editing; methodology: Jean de Dieu H. Tuyishime.Conceptualization; writing—original draft; methodology; writing—review and editing: Francois Xavier Sunday. Methodology; validation; writing—original draft; writing—review and editing: Jeanine Ahishakiye.

## CONFLICT OF INTEREST STATEMENT

Authors declare that they have no conflicts of interest.

## FUNDING INFORMATION

This research did not receive any funding.

## ETHICS STATEMENT

This study utilized publicly available secondary data requested from the Demographic and Health Surveys (DHS) program. The DHS program ensures strict adherence to ethical guidelines and protocols in data collection, protecting the privacy and confidentiality of survey participants. As the present study is based on de‐identified data, no additional ethical approval was required.

## Data Availability

The datasets of the current study are available from the corresponding author on request.
